# Population-based estimates of humoral autoimmunity from the U.S. National Health and Nutrition Examination Surveys, 1960–2014

**DOI:** 10.1371/journal.pone.0226516

**Published:** 2020-01-13

**Authors:** Charles F. Dillon, Michael H. Weisman, Frederick W. Miller

**Affiliations:** 1 National Institute of Environmental Health Sciences, NIH, Bethesda, Maryland, United States of America; 2 Cedars-Sinai Medical Center, David Geffen School of Medicine at UCLA, Los Angeles, CA, United States of America; Stony Brook University, Graduate Program in Public Health, UNITED STATES

## Abstract

**Objective:**

Based on US National Health and Nutrition Examination Survey (NHANES) data, we attempted to provide an unbiased, population-based estimate of autoantibody prevalence overall and by age and sex.

**Methods:**

US autoantibody prevalence estimates for detectable rheumatoid factor, anti-thyroglobulin, anti-thyroperoxidase, anti-transglutaminase, anti-endomysial, anti-GAD65, antinuclear autoantibodies, and autoantibodies to extractable nuclear antigens were estimated from the 1960–1962 National Health Examination Survey, NHANES III (1988–1994), and the NHANES 1999–2014 cross-sectional surveys. Survey design variables and sample weights were used to account for differential probabilities of selection within the complex survey design. Data analysis used SAS^TM^ and SUDAAN^™^ software. US Census Bureau data were used to estimate the absolute numbers of persons with autoantibodies.

**Results:**

NHANES III data show that the overall US prevalence of having a detectable serum autoantibody is substantial in adults, in both women and men. Thyroid autoantibodies were present in 18% of US adults (31 million persons) including 10% of younger adults and 25% of older persons. Overall autoantibody prevalences increased significantly with age: 32% of US adults 60+ years of age (12.8 million persons) had at least one of the four autoantibodies rheumatoid factor, anti-thyroglobulin, anti-thyroperoxidase, or anti-tissue transglutaminase. Older women had higher levels of autoantibodies, but this was a relative difference. Autoantibody prevalence in both sexes was substantial (women 39%; men 22%). Fourteen percent of adults 60+ years of age have multiple autoantibodies.

**Conclusions:**

Autoantibodies are present in a significant fraction of the general population, especially in older adults and women relative to men. Although all known clinically significant autoantibodies were not analyzed, these data provide an important population perspective on the scope and magnitude of humoral autoimmunity in the US. This is vital for prevention efforts to reduce autoimmune disease and helps clarify the potential impact of autoimmunity on the general population.

## Introduction

Although the presence of an autoimmune serological marker does not always indicate clinical autoimmune disease (AID), it does mark the presence of biologic autoimmunity. Autoantibodies with target organ specificity have significant predictive value because they represent a risk factor for the development of a specific AID or phenotype. Since autoantibodies can be detected in the prodromal phase of AID development, they are potentially useful for detecting treatable early disease [1]. Although some autoimmune serologic markers appear transiently after apparently self-limited infections, immunizations, or injuries, in many situations autoantibodies are also clearly pathogenic [2–9].

In clinical practice and medical research studies, the significance of a single autoantibody or a small set of related autoantibodies is typically emphasized, and studying them has been a proven, productive model for pathophysiologic investigations since the late 1940s. However, this clinical “single-antibody” approach might also have served to obscure the overall magnitude, scope, and biologic significance of autoimmunity. The tendency to investigate autoantibodies and AIDs on a one-by-one basis might have inadvertently created a widespread assumption that autoimmune disorders are isolated examples of clinical problems that primarily affect only a minority of persons.

Epidemiological research, however, has shown that autoimmune disorders are common, have genetic links among one another, and can overlap or even evolve from one clinical phenotype to another [10–13]. An estimated 3–7% of the general population has an AID, depending on the specific AIDs studied [10, 11, 14]. The population prevalence of autoantibodies occurring solely within recognized AIDs is therefore substantial, with potentially millions of persons affected. Autoantibodies are also increasingly identified as pathogenic in new ways beyond their role in classic AIDs. For example, autoantibodies have been identified as a potential cause of heart or lung disease in adults and may participate in the process of atherosclerosis [15–21]. A number of maternal autoantibodies show person-to-person transmission of disease to the developing fetus during pregnancy and may have adverse effects, the most outstanding is of these being neonatal lupus, neonatal thyroid disease and thyroid autoantibody related early fetal loss and pre-term birth [22–27]. The above findings have significant implications for clinical and public health efforts to control AID.

Many clinical illnesses, including AID, normally exist within a spectrum from very mild to severe disease, which is sometimes even fatal. Typically, mild and moderate cases far outnumber severe cases (e.g., hypertension and diabetes). Although a fraction of mild cases might never progress, clinical and public health surveillance of the full spectrum of AID disease is essential, because a significant fraction of patients progress from preclinical illness (positive autoantibodies or biomarkers only) to subclinical illness (few or no clinical symptoms but positive autoantibody, laboratory, and/or imaging studies) to overt, classically active disease [28, 29]. Individuals with preclinical, autoantibody-positive disease have been shown recently to be at high risk of developing AID such as family members of AID cases,[30], and clinical prevention trials in rheumatoid arthritis and autoimmune diabetes are currently addressing this concern [31–35]. Most cases of subclinical AID and many cases of mild AID are not currently detected; in the general population, the number of persons at risk of AID and those with undiagnosed AID may be large. In addition, there is currently incomplete surveillance of AID-related mortality of persons with diagnosed AID even though published estimates, based on the available data, reveal that AIDs are a leading cause of death among adult women in the United States [36]. From a population perspective, it is essential to have an accurate assessment of the overall prevalence of humoral autoimmunity because it provides one type of upper bound for the magnitude of the population prevalence of autoimmunity.

Autoantibodies can also be sensitive markers of exposure to environmental causes of AID, such as AID caused by prescription drugs or exposure to dietary gluten that causes celiac disease [37, 38]. Currently additional chemical exposures are under investigation as potential causes of AID [39, 40]. Since autoantibodies usually have much higher population frequencies than the AIDs linked to them, they offer the opportunity for larger, more adequate epidemiologic studies of potential causal exposures. Attention to humoral autoimmunity, its prevalence, causes, and triggers is therefore essential to primary prevention efforts to reduce the incidence of AID [41, 42]. Surveillance and detection of autoantibodies are key to secondary disease-prevention efforts, i.e., public health disease-monitoring programs for early detection of disease and clinical treatment to prevent irreversible target organ complications or fatalities [43]. These efforts are important because, although medical therapy does exist for AID, cures are not at hand, and presently many patients face a lifetime of chronic illness and burdensome treatments.

To support such prevention efforts, we aim to provide an overall US population-based estimate for the prevalence of humoral autoimmunity. The US National Health and Nutrition Examination Survey (NHANES) has collected nationally representative health examination and laboratory data for over 50 years. Although NHANES has not fielded a complete set of serum autoantibodies, substantial data currently exist (Table 1). We assessed existing data from NHANES III 1988–1994 to determine the overall general population prevalence of serological autoimmunity, as this NHANES survey cycle has the most complete panel of autoantibody tests. Additional autoantibody data from the US National Health Examination Survey (NHES I) 1960–1962 and the more current NHANES 1999–2014 are also analyzed to provide a more comprehensive perspective.

**Table 1:** US National Health & Nutrition Examination Survey autoantibody Data, 1960–2014.

Autoantibodies	Disease Associations	1960–1962	1988–1994	1999–2004	2007–2008	2009–2012	2013–2014
Rheumatoid Factor	Rheumatoid Arthritis SLE MCTD	**●**	**●**				
ANA	SLE SS SJS MCTD PBC AIH DM PM			**●**			
ENA Autoantibodies	SLE SS SJS MCTD PM DM			**●**			
Anti-Endomysial	Celiac Disease		**●**			**●**	**●**
Anti-Trans-Glutaminase	Celiac Disease		**●**			**●**	**●**
Anti-GAD65	Autoimmune Diabetes		**●**				
Anti-Thyroperoxidase	Autoimmune Thyroiditis		**●**	**●**	**●**	**●**	
Anti-Thyroglobulin	Autoimmune Thyroiditis		**●**	**●**	**●**	**●**	

Abbreviations: ANA = Anti-Nuclear Autoantibodies by immunofluorescence; ENA = Extractable Nuclear Antigen; GAD65 = 65kDa isoform of glutamic acid decarboxylase. Disease acronyms: AIH: autoimmune hepatitis; DH: dermatitis herpetiformis; DM: dermatomyositis; MCTD: mixed connective tissue disease; PBC: primary biliary cholangitis; PM: Polymyositis; SJS: Sjogren's syndrome; SLE: systemic lupus erythematosus; SS: systemic sclerosis. Note: ANA is a general autoimmunity screening test. If positive, it is usually followed up with testing for specific autoantibodies.

The NHANES autoantibody data were collected primarily to fulfill case classification criteria for studies that estimated the US prevalence of autoimmune and rheumatic diseases, such as rheumatoid arthritis, thyroid disorders, diabetes, and celiac disease; hence, the NHANES autoantibody data relate directly to common AIDs [44–48]. These studies were performed mainly in adult populations, but used different target age ranges. Nevertheless, while the NHANES autoantibody data are incomplete, they can potentially provide an estimate of the overall US population prevalence of a key set of clinically relevant serum autoantibodies. Specifically, the NHANES data include the organ-specific autoantibodies anti-thyroglobulin (anti-TG), anti-thyroperoxidase autoantibodies (anti-TPO), autoantibodies to the 65-kDa isoform of glutamic acid decarboxylase (anti-GAD65), anti-tissue trans-glutaminase (anti-TTG), and anti-endomysial autoantibodies (anti-EMA). The data also include autoantibodies related to systemic diseases, such as rheumatoid factor (RF) as well as antinuclear autoantibodies (ANA) and antibodies to extractable nuclear antigens (anti-ENA) (Table 1**)**. Basic US prevalence data for some of the NHANES autoantibodies are currently published [44–49]; however, other NHANES autoantibody data remain unanalyzed and unreported. We assembled and systematically analyzed all existing NHANES autoantibody data to address the following questions:

1. What is the overall prevalence of detectable autoantibodies in the US population?
2. What is the true population-based demographic distribution of humoral autoimmunity according to age and sex? Is humoral autoimmunity more common among women, especially younger adult women?
3. On a general population level, how common is it for individuals to have single, isolated autoantibodies or multiple autoantibodies? On a population level, is there evidence that specific autoantibodies are associated with one another?

## Methods

### NHANES data collections

Since the 1960s, NHES/NHANES has conducted cross-sectional surveys that monitor the health and nutritional status of noninstitutionalized civilians in the US. The sampling frame does not include hospitalized persons or those in long-term care facilities. Each NHANES sample population is considered nationally representative. Data are collected via household interviews as well as standardized physical examinations, and biological specimen collections are performed in specially equipped mobile examination centers. The NHANES surveys are demographically based, and samples are selected through a complex, multistage, survey design [50, 51]. The earliest NHANES surveys were designed solely to estimate national-level prevalences, whereas recent surveys also employed oversampling strategies to obtain sufficient data to study major demographic subgroups. NHANES survey response rates have historically been held above a benchmark of 70% [52]. The NHANES survey protocols, survey research conduct, and data release are reviewed and approved by the US National Center for Health Statistics Ethics Review Board. Written informed consent for data collection was obtained from all subjects. This study is based on publicly available data from the NHANES website [53]. For this report we used publicly available data from the US National Health & Nutrition Examination Survey website which is accessible at this internet link: https://wwwn.cdc.gov/nchs/nhanes/. On this webpage, data is organized by survey calendar year. Clicking on the icon for each year opens up a new webpage with links to all the available questionnaire, laboratory and health examination data for that specific survey cycle. Each individual public release dataset also has documentation explaining the available variables and analytic guidelines. We did not use any additional special data besides the NHANES public release data for this report.

### Demographic and laboratory data

Demographic data, including the respondent's age and sex, were collected during the household interview. In the tables presented herein, age ranges for which no NHANES data were collected are indicated. To provide a set of standardized and comparable age groups with sufficient sample sizes to reliably estimate autoantibody prevalence, we created four adult age groups (18–24 years, 25–39 years, 40–59 years, and 60+ years) [52]. The analyses presented here include autoantibody prevalence data from the NHES I 1960–1962, NHANES III 1988–1994, and NHANES 1999–2014. New autoantibody prevalence estimates for RF were calculated, as they had not been published for the NHANES III 1988–1994 data, and modern statistical software for complex survey analysis was not available when the NHES I 1960–1962 RF prevalence estimates were originally reported [54].

The laboratory methods and quality control procedures used for autoantibody analysis are described on the NHANES Data and Documentation website and in published articles. In brief, for NHANES III 1988–1994, the two thyroid autoantibodies (anti-TPO and anti-TG) were measured by using a highly sensitive, direct radioimmunoassay system (Kronus, San Clemente, CA) [55, 56]; in NHANES 2001–2002 and 2007–2012, the same two thyroid autoantibodies were measured by sequential two-step immunoenzymatic sandwich assays [57, 58]. The celiac autoantibody (anti-TTG) was measured by enzyme-linked immunosorbent assay (ELISA) for semi-quantitative detection of immunoglobulin A antibodies to tissue transglutaminase in human serum; anti-EMA testing was performed by indirect immunofluorescence using cryostat-prepared sections of rhesus monkey esophagus as substrate [59, 60]. Autoantibodies to anti-GAD65 were detected by immunobinding of serum with in vitro transcribed/translated 35S-methionine–labeled recombinant human GAD65, using the method described by Grubin et al. [47, 61, 62]. ANA in serum samples were evaluated by standard immunofluorescence ANA testing using commercial HEp-2 ANA slides (Inova Diagnostics). ANA immunofluorescent (ANA-positive) sera were tested by immunoprecipitation of 35S-methionine–labeled K562 cell extracts to determine specific autoantibodies [63, 64]. Rheumatoid factor in the NHES I 1960–1962 survey cycle was tested at the laboratories of the National Institute of Arthritis & Metabolic Diseases using the bentonite flocculation method [54, 65], and in the NHANES III 1988–1994 survey, it was measured using the Singer-Plotz latex agglutination test [66]. NHANES III RF specimens were screened using latex-enhanced nephelometry prior to obtaining titers.

Reference ranges to calculate overall prevalence estimates of detectable autoantibodies were as follows: for the NHANES III 1988–1994 data, the normal range for anti-TG was < 1.0 IU/ml and for anti-TPO it was < 0.5 IU/ml [45, 55, 56]; for NHANES 2007–2012, the normal ranges were anti-TG < 0.4 IU/ml and anti-TPO <0.9 IU/ml [57, 58]. For anti-TTG, test results were considered negative if < 4.0 U/ml, weakly positive if 4–10 U/ml, and positive if >10 U/ml. For anti-EMA, a result was considered positive if fluorescence was observed at a dilution ratio ≥ 1:5 [48, 59, 60]. Anti-EMA testing was performed only if anti-TTG was positive. For anti-GAD65, the cutoff point for the assay was the 99th percentile of anti-GAD65 levels, calculated from a laboratory reference distribution [47]. For ANA, immunofluorescence staining intensities were graded on a 0–4 scale using a standard reference gallery with staining signal intensities of 3 and 4 considered positive. Anti-ENA testing by standard immunoprecipitation assay was performed only in ANA-positive specimens [49, 63, 67]. NHANES RF data were reported in titers depending on the method used: in the 1960–1962 survey, a positive RF was a titer of 1:32 [54]; for NHANES 1988–1994, a titer of 1:20 [66]. Autoantibody titer distributions for NHES I and NHANES III RF data are presented in S1 Fig. The population prevalence of a high-titer RF was defined here according to American College of Rheumatology/European League Against Rheumatism guidelines for high RF in rheumatoid arthritis—values 3 times greater than the upper limit of normal for the laboratory assay [68]. For the NHES I 1960–1962 and the NHANES III 1988–1994 RF data, high titer RF cut points were ≥ 1:128 and ≥ 1:160, respectively. In the absence of consensus, prevalences for higher-level NHANES III thyroid autoantibodies were calculated as ≥ 95^th^ percentile of a log-transformed distribution.

### Statistical analysis

NHANES design variables and health examination sample weights were used to account for differential probabilities of selection within the complex NHANES sample design as well as to obtain prevalence estimates (± standard errors) representative for the noninstitutionalized US population. The sample weights account for the unequal selection probabilities of subgroups, adjusted for nonresponse and noncoverage. Dataset assembly was performed using SAS^TM^ (Release 9.4, SAS Institute, Inc., Cary, NC), and statistical analyses were performed using SAS^TM^ and SUDAAN^™^ (Release 11.0.1; Research Triangle Institute, Research Triangle Park, NC). Age-stratified autoantibody prevalences were calculated with age groups constructed to maximize subgroup sample sizes; absolute US population counts were estimated in deciles. Age-adjusted prevalences were computed by direct standardization to US Census Bureau estimates for the US civilian, noninstitutionalized population [52]. Standard errors were estimated by Taylor series linearization. The equality of prevalence estimates for autoantibodies was tested (univariately) at the α = 0.05 level using a Student’s *t* statistic with the appropriate degrees of freedom. Age-trend testing was performed using orthogonal contrast matrices [69]. NHANES III analytic guidelines were used to set criteria for minimum acceptable sample sizes (based on design effect, degrees of freedom, specified proportions), and relative standard errors (RSE) were used to assess statistical stability of computed estimates [70]. Estimates with RSE ≥ 30%, those based on less than 12 degrees of freedom, or on sample sizes less than recommended are designated in tables as potentially unreliable and should be interpreted with caution. Confidence intervals for these are not presented.

Anti-GAD65 data were originally collected as a small, special purpose non-representative case-control study. The standard NHANES III sample weights were reweighted to estimate national-level anti-GAD65 prevalences using SUDAAN PROC WTADJUST. NHANES III survey autoantibody sample sizes varied due to sera availability. For estimating overall combined autoantibody prevalences in the 60+ year age group, a total of 5,302 sample persons (63% of those initially screened) were examined. 4,965 had blood available for RF analysis (safety exclusions, difficult blood draw, small blood volume, phlebotomy refusals). Anti-TG and anti-TPO were analyzed in a later second round of laboratory testing (sample = 4,835). Anti-TTG was analyzed some 15 years later using a set of stored sera samples (sample = 4,312). 4,805 sample persons had all three high frequency autoantibodies tested (RF, anti-TG, anti-TPO); 4,243 survey participants had four autoantibodies tested (RF, anti-TG, anti-TPO, anti-TTG). A sensitivity analysis was performed to assess potential effects of missing data. Because the prevalence of anti-TTG was very low, the population prevalence of the three antibodies RF, anti-TG and anti-TPO was equivalent to and not statistically different from the prevalence of the four antibodies RF, anti-TG, anti-TPO and anti-TTG. Also, there was no reduction in sample degrees of freedom or systematic bias that would require sample reweighting for a combined four-antibody analysis. For the sake of completeness in antibody coverage, the statistical analyses for overall US autoantibody prevalence presented here is the four-antibody combined antibody prevalence estimate analyzed as a subdomain of the full sample dataset (SAS Nomcar option). Standard survey weights were employed that adjust for demographic coverage and nonresponse at the examination level.

## Results

Tables 2 and 3 present NHANES’ US national prevalence estimates and distributions by age for seven autoantibodies plus ANA testing. Age ranges in which no autoantibody data were collected are indicated. The results reveal high prevalences of target organ autoantibodies and systemic autoantibodies. The NHANES III 1988–1994 survey data show that thyroid autoantibodies were particularly common in the US adult population (Table 2). This is especially true for anti-TG and anti-TPO autoantibodies, which have overall prevalences of 12.1% and 13.9%, respectively, among adults 18+ years. Clear age trends are evident in the NHANES III thyroid autoantibody data: the population prevalence of anti-TG autoantibody is 5.9% (95% CI 4.2–7.6%) among 18–24-year-olds and 18.1% (95% CI 16.7–19.5%) for those 60+ years of age. For anti-TPO, prevalences in the same age groups are 7.1% (95% CI 5.7–8.5%) and 21.1% (95% CI 19.5–22.8%), respectively. Statistical testing shows a significant linear trend with age separately for each thyroid autoantibody (*P* < .01). The more recent NHANES 2007–2012 thyroid autoantibody testing also showed high prevalences of anti-TG and anti-TPO autoantibodies: the overall US population prevalence of anti-TG for adults 18+ years old is 7.7% (95% CI 6.9–8.5%), and overall anti-TPO prevalence is 11.7% (95% CI 10.5–12.8%). These prevalences are substantial and are similar to the NHANES III estimates, but both were statistically significantly lower than those seen in the NHANES III anti-TG and anti-TPO data, respectively (*P* < .01). In the NHANES 2009–2012 data on thyroid autoantibodies, increasing prevalence with age was also seen: the population prevalence of anti-TG was 5.7% (95% CI 3.9–7.4%) among 18–24-year-olds and 11.2% (95% CI 9.3–13.2%) for those 60+ years of age; for anti-TPO, these prevalences were 6.3% (95% CI 4.4–8.2%) and 15.1% (95% CI 13.3–16.8%), respectively (age-trend tests both *P* < .01).

**Table 2:** US population prevalence of thyroid autoantibodies and rheumatoid factor, 1960–2012.

	**Anti-Thyroglobulin Ab**			**Anti-Thyroperoxidase Ab**			**Rheumatoid Factor**		
	**NHANES III (1988–1994)**			**NHANES III (1988–1994)**			**NHES I (1960–1962)**		
**Target Age (y)**	**18+ years**			**18+ years**			**18–79 years**		
**Prevalence**	**N**	**%**	**95%CI**	**N**	**%**	**95%CI**	**N**	**%**	**95%CI**
Overall	15,956	12.1	11.0–13.2	15,956	13.9	13.1–14.7	6,468	3.1	2.5–3.6
18–24	2,286	5.9	4.2–7.6	2,286	7.1	5.7–8.5	920	0.7	0.1–1.4
25–39	4,674	10.3	8.2–12.4	4,674	11.1	9.3–12.9	2,123	1.8	1.2–2.4
40–59	4,161	12.8	11.2–14.4	4,161	15.2	14.0–16.5	2,364	3.6	2.5–4.7
60+	4,835	18.1	16.7–19.5	4,835	21.1	19.5–22.8	1,061	5.8	4.5–7.0
	**Anti-Thyroglobulin Ab**			**Anti-Thyroperoxidase Ab**			**Rheumatoid Factor**		
	**NHANES 2007–2012**			**NHANES 2007–2012**			**NHANES III (1988–1994)**		
**Target Age (y)**	**18+ years**			**18+ years**			**60+ years**		
**Prevalence**	**N**	**%**	**95%CI**	**N**	**%**	**95%CI**	**N**	**%**	**95%CI**
Overall	9,915	7.7	6.9–8.5	9,131	11.7	10.5–12.8	nd	nd	nd
18–24	1,160	5.7	3.9–7.4	1,161	6.3	4.4–8.2	nd	nd	nd
25–39	2,162	4.7	3.6–5.7	2,150	8.3	6.7–9.8	nd	nd	nd
40–59	2,819	8.2	6.4–10.0	2,804	13.6	11.3–15.9	nd	nd	nd
60+	3,014	11.2	9.3–13.2	3,016	15.1	13.3–16.8	5,270	6.5	5.9–7.1

Notes: Data collection target age ranges vary among NHANES studies: nd indicates data not collected. Abbreviations: Ab = antibody; y = years; NHANES = National Health and Nutrition Examination Survey; NHES = National Health Examination Survey; N = total sample; % = prevalence %; 95%CI = 95% confidence interval; anti-TG = anti-thyroglobulin; anti-TPO = anti-thyroid peroxidase; RF = rheumatoid factor.

**Table 3:** US prevalence of celiac, diabetes and antinuclear autoantibodies, 1988–2014.

	**Anti-TTG**			**Anti-GAD65**			**Anti-Nuclear Ab**		
	**NHANES III (1988–1994)**			**NHANES III (1988–1994)**			**NHANES 1999–2004**		
**Target Age (y)**	**50+ years**			**40+ years**			**18+ years**		
**Prevalence**	**N**	**%**	**95%CI**	**N**	**%**	**95%CI**	N	**%**	**95%CI**
Overall	nd	nd	nd	nd	nd	nd	3,863	14.2	12.4–15.9
18–24	nd	nd	nd	nd	nd	nd	654	14.3	9.5–19.1
25–39	nd	nd	nd	nd	nd	nd	972	12.8	9.8–15.8
40–59	1,554	0.7	*	775	2.0	0–4.1	1,060	13.9	11.1–16.8
60+	4,485	1.5	0.9–2.2	1,325	3.1	0–6.1	1,177	16.5	13–19.9
	**Anti-TTG**			**Anti-EMA**			**Anti-ENA**		
	**NHANES 2009–2014**			**NHANES 2009–2014**			**NHANES 1999–2004**		
**Target Age (y)**	**18+ years**			**18+ years**			**18+ years**		
**Prevalence**	**N**	**%**	**95%CI**	**N**	**%**	**95%CI**	**N**	**%**	**95%CI**
Overall	16,667	0.8	0.6–1.0	16,667	0.5	0.3–0.6	3,863	1.1	0.7–1.6
18–24	2,280	0.8	*	2,280	0.5	*	654	0.7	*
25–39	3,975	0.8	0.4–1.3	3,975	0.7	0–1.4	972	1.1	*
40–59	5,332	0.8	0.5–1.2	5,332	0.6	0.1–1.0	1,060	1.3	*
60+	5,080	0.6	0–1.2	5,080	0.2	*	1,177	1.0	*

Notes: Data collection target age ranges vary among NHANES studies: nd indicates data not collected. For NHANES III anti-TTG, results in the 40-59-year age category are estimates for adults 50–59 years as only data for adults 50+ years were collected.

*Weighted estimate not statistically reliable by National Center for Health Statistics criteria; confidence interval estimates not shown.

Abbreviations: Ab = antibody; y = years; NHANES = National Health and Nutrition Examination Survey; N = total sample; % = prevalence %; 95%CI = 95% confidence interval; TTG = tissue transglutaminase; GAD-65 = antibodies to the 65-kDa isoform of glutamic acid decarboxylase; Pctile = percentile; IF = immunofluorescence; EMA = endomysial autoantibodies; ENA extractable nuclear antigen.

RF was studied in the NHES I 1960–1962 and in the NHANES III 1988–1994 surveys. The NHES I was the only NHANES survey to have measured RF across all adult age ranges. Those data (Table 2) show a clear trend of increasing RF prevalence by age (trend test *P* < .01): younger adults had the lowest prevalence, and the oldest adults had the highest prevalence (0.7% at 18–24 years vs. 5.8% at 60+ years). Almost three decades later, NHANES III measured RF again, but only as part of an assessment of rheumatoid arthritis prevalence in adults aged 60 years and older. Remarkably, RF prevalence estimates for the ≥60-year-old age group in the NHES I data and in the NHANES III data are not significantly different from one another (5.8% [95% CI 4.5–7.0%] vs. 6.5% [95% CI 5.9–7.1%], respectively; *P* = .33). The RF prevalences in NHANES III were 5.3% (95% CI 4.1–6.5%) for those aged 60–69 years, 7.8% (95% CI 6.1–9.6%) for those aged 70–79 years, and 7.5% (95% CI 6.1–8.8%) in those 80+ years, but age-trend testing among older adults was not significant.

Celiac disease-related autoantibodies anti-TTG and anti-EMA were studied in adults 50 years of age and older in NHANES III and all adult age ranges in NHANES 2009–2014 (Table 3). In the NHANES III data, the 50–59-year-olds did not have an adequate sample size for reliable statistical estimation; however, among adults 60+ years of age, 1.5% (95% CI 0.9–2.2%) tested positive for anti-TTG autoantibodies. In the NHANES 2009–2014 survey data, 0.8% (95% CI 0.6–1.0%) of adults aged 18+ years tested positive for anti-TTG, and 0.5% (95% CI 0.3–0.6%) tested positive for anti-EMA autoantibodies. Sample sizes in the NHANES 2009–2014 survey were not adequate to test for age trends for anti-TTG and anti-EMA. Autoantibodies to anti-GAD65 were studied in a small subsample of NHANES III participants aged 40+ years; 2.0% of those aged 40–59 years and 3.1% of those aged 60+ years tested positive for anti-GAD65. Finally, in NHANES 1999–2004, ANA with follow-up testing for specific anti-ENA immunoprecipitants was performed; 14.2% of adults sampled screened positive for ANA, and among them 1.1% (95% CI 0.7–1.6%) tested positive for anti-ENA. The ANA data for adults did not show age-related trends. Sample sizes did not support reliable testing for age trends in anti-ENA.

**Fig 1:**
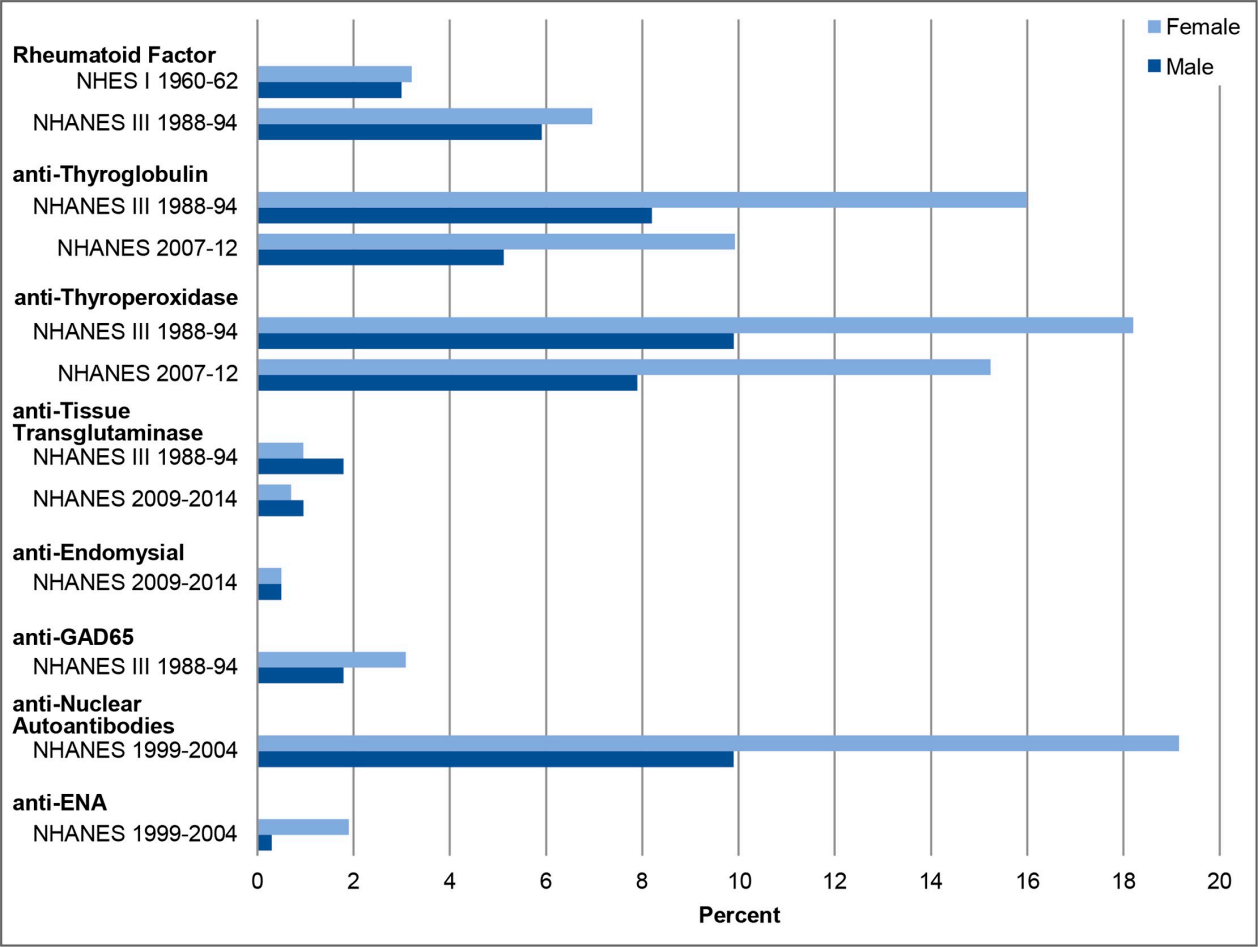
US prevalences of autoantibodies by sex, NHANES 1960–2014. Abbreviations: F/M Ratio = weighted female to male prevalence ratio; GAD-65 = antibodies to the 65-kDa isoform of glutamic acid decarboxylase; ENA = Extractable Nuclear Antigen.

Table 4 and Fig 1 present age-adjusted autoantibody prevalence rates by sex. For anti-TG and anti-TPO, the most common autoantibodies, women 18+ years had a two-fold higher prevalence than men in both NHANES III 1988–1994 and NHANES 2007–2012 survey data. These differences were significant. Female predominance is also clearly seen in overall ANA testing by immunofluorescence, which also shows an approximate two-fold higher prevalence among women. Conversely, there were no significant differences between women and men for the two celiac disease-related autoantibodies, anti-TTG, and anti-EMA, in either the NHANES III 1988–1994 or the NHANES 2007–2012 data. Further, there were no significant differences between women and men in the prevalence of RF in the NHES I 1960–1962 or NHANES III 1988–1994 data. Analysis of potential sex-related prevalence differences for anti-GAD65 and age-trend testing for anti-GAD65 and anti-ENA autoantibodies was not performed due to low antibody prevalences and small sample sizes.

**Table 4:** US autoantibody prevalence by sex, NHANES 1960–2014.

		Women			Men			F/M	
Autoantibody	Age	N	%	95%CI	N	%	95%CI	Ratio	*P*
Rheumatoid factor									
NHES I 1960–62	20+	3,322	3.4	2.7–4.3	2,881	3	2.3–3.9	1.1	0.45
NHANES III 1988–94	60+	2,713	7	6.1–8.1	2,557	5.9	5.0–7.0	1.2	0.22
Anti-thyroglobulin									
NHANES III 1988–94	20+	8,139	16	14.8–17.7	7,174	8.2	6.9–9.7	2	< .01
NHANES 2007–12	20+	4,415	9.9	8.4–11.4	4,313	5.4	4.4–6.4	1.8	< .01
Anti-thyroperoxidase									
NHANES III 1988–94	20+	8,139	18.2	17.2–19.2	7,174	9.9	8.8–11.2	1.8	< .01
NHANES 2007–12	20+	4,389	15.5	14.0–17.0	4,314	7.8	6.4–9.1	2	< .01
Anti-tissue transglutaminase									
NHANES III 1988–94	50+	3,187	0.8	0.3–1.3	2,847	1.8	1.1–2.6	0.4	0.03
NHANES 2009–14	20+	8,134	0.7	0.4–1.0	7,695	0.8	0.6–1.0	0.9	0.51
Anti-endomysial									
NHANES 2009–14	20+	8,134	0.5	0.2–0.7	7,696	0.5	0.4–0.7	1	0.77
Anti-GAD65									
NHANES III 1988–94	40+	1,083	2.9	1.5–4.3	1017	1.8	*	*	*
Anti-nuclear autoantibodies									
NHANES 1999–04	20+	1,892	18.7	16.0–21.3	1,674	9.9	7.8–11.9	1.9	< .01
Anti-ENA									
NHANES 1999–04	20+	1,892	1.9	1.2–2.9	1,674	0.3	*	*	*

*Variance estimate statistically unreliable, statistical estimates not shown.

Abbreviations: Age: age in years; N = total sample; % = prevalence; 95%CI = 95% confidence interval; *P* = P value for t test of difference between male and female prevalences; F/M Ratio = weighted female to male prevalence ratio; NHES = National Health Examination Survey; NHANES = National Health and Nutrition Examination Survey; GAD-65 = antibodies to the 65-kDa isoform of glutamic acid decarboxylase; ENA = extractable nuclear antigen.

### Overall US autoantibody prevalence estimates

**Fig 2:**
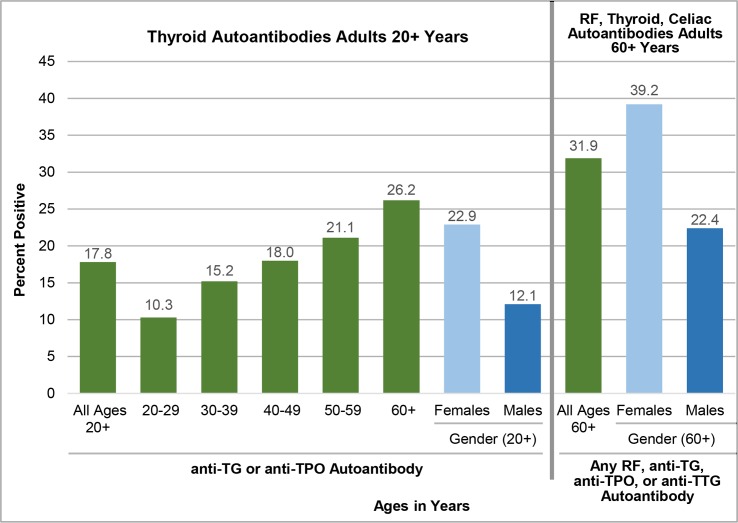
The prevalence of persons with a detectable serum autoantibody, NHANES 1988–1994. Abbreviations: RF = Rheumatoid Factor; TG = Thyroglobulin; TPO = Thyroid Peroxidase; TTG = Tissue Transglutaminase.

Table 5 and Fig 2 present overall age- and sex-specific prevalences by deciles for the detection of any of the four serum autoantibodies (anti-TG, anti-TPO, anti-TTG, and RF), based on NHANES III data. Section a in Table 5 presents combined prevalence estimates for the thyroid autoantibodies anti-TG and anti-TPO, which were the only autoantibodies studied in a full sample of adults 20+ years and older; section b shows the overall combined autoantibody prevalences, i.e., the prevalence of an individual having any one of the four autoantibodies—RF, anti-TG, anti-TPO, or anti-TTG—among adults aged 60+ years. For adults aged 18+ years, the prevalence of having a detectable anti-TG or anti-TPO or both was 17.8% (95% CI 16.9–18.8%) (Table 5). There is also a significantly higher overall thyroid autoantibody prevalence with older age (*P* < .01). Although women had a two-fold higher thyroid autoantibody prevalence than men, combined thyroid autoantibody prevalences are substantial in both sexes (22.9% among women vs. 12.1% among men). Further, the prevalence of having any of the three autoantibodies anti-TG, anti-TPO or RF in the NHANES III data substantial: this overall prevalence is 30.9% (95%CI 29.4–32.3); women had a prevalence of 38.5% (95%CI 35.9–41.0) and men 20.8% (95% CI 18.5–23.1).

**Table 5:** US prevalence of a detectable serum autoantibody, NHANES 1988–1994.

**a. Thyroid Autoantibodies** **Anti-TG or anti-TPO Ab**	**US Population** **(millions)**	**N**	**% Positive**	**95%CI**	**N Positive** **(millions)**	**95%CI** **(millions)**
All ages (20+ Years)	177.2	15,313	17.8	16.9–18.8	31.5	29.9–33.3
20–29 years	39.2	3,237	10.3	8.3–12.2	4.0	3.3–4.8
30–39 years	42.2	3,080	15.2	12.4–18.0	6.5	5.3–7.7
40–49 years	33.2	2,422	18.0	16.0–20.0	6.0	5.3–6.6
50–59 years	22.0	1,739	21.1	18.6–23.5	4.6	4.1–5.2
60+ years	40.1	4,835	26.2	24.4–27.9	10.5	9.8–11.2
Sex (20+ years)						
Females	92.8	8,139	22.9	21.8–23.9	21.3	20.2–22.2
Males	84.4	7,174	12.1	10.8–13.6	10.2	9.0–11.4
**b. Any RF, anti-TG, TPO or TTG Autoantibody**						
Age 60+ years	40.1	4,243	31.9	30.3–33.5	12.8	12.1–13.4
Sex (60+ years)						
Females	23.0	2,195	39.2	36.6–41.8	9.0	8.4–9.6
Males	17.1	2,048	22.4	19.9–25.1	3.8	3.4–4.3

Population estimates from US census data. Abbreviations: anti-TG = anti-thyroglobulin; anti-TPO = anti-thyroperoxidase; Ab = antibody; 95%CI = 95% Confidence Interval; N = sample size for estimates; RF = rheumatoid factor; TTG = anti-tissue transglutaminase.

Table 5 section b shows overall autoantibody prevalence estimates for adults 60+ years of age. The prevalence of having at least one of the four autoantibodies (RF, anti-TG, anti-TPO, or anti-TTG) was 31.9% (95% CI 30.3–33.5%). In adults 60+ years old, there is a trend of increasing autoantibody prevalence with age. For example, in 60–69-year-olds, the prevalence of having any one of the four autoantibodies was 29.3% (95% CI 27.0–31.6%; *P* < .01), and in the 70+-year age group it was 35% (95% CI 32.7–37.3%; *P* < .01). Remarkably, almost 40% of older women and almost 22% of older men had one of the four autoantibodies. Finally, to underscore that autoantibodies are highly prevalent in the general US population, absolute population counts in millions corresponding to the autoantibody prevalence estimates are presented. This analysis shows that approximately 31.5 million US adults aged 20+ years (95% CI 29.9–33.3 million) had one of the two thyroid autoantibodies, whereas 12.8 million older US adults (95% CI 12.1–13.4 million) aged 60+ years had one of the four autoantibodies—RF, anti-TG, anti-TPO, or anti-TTG.

### Population prevalences of single (isolated) and multiple autoantibodies

A common clinical question is, “what is the likelihood for a person to test positive for a single autoantibody or multiple autoantibodies?” We estimated the US population prevalence of a specific individual having a single isolated autoantibody, as well as the prevalence of an individual having multiple autoantibodies in adults aged 60 years and older using the RF, anti-TG, anti-TPO, and anti-TTG data from NHANES III (S1 Table). The probability of detecting a single, isolated autoantibody in an individual 60+ years was 17.6% (95%CI 16.1–19.2): 4.5% of the population had anti-TG as an isolated finding, 7.1% had anti-TPO, 1.3% had anti-TTG, and 4.7% had an isolated RF. An estimated 14.5% (95%CI 13.1–15.9) of older adults have multiple autoantibodies (two or more of the four autoantibodies tested). The most prevalent autoantibody combination is anti-TG and anti-TPO (12.5%). All other two- and three-autoantibody combinations are uncommon (generally less than 1% prevalence). Two exceptions are the combinations that include anti-TPO: anti-TPO and RF or anti-TG, anti-TPO, and RF. Only a single individual in the dataset had all four autoantibodies detected at the same time.

Finally, the Supporting Information also includes tables and figures relevant to interpreting NHANES anti-TG, anti-TPO, and RF autoantibody data. S2 Table presents age-stratified RF autoantibody prevalences separately by sex, to demonstrate that age-related increases in RF occur in both sexes. A previous detailed analysis demonstrated age-related increases in anti-TG and anti-TPO in both sexes [45]. S3 Table presents estimates for the prevalence of high-level autoantibodies. S1 Fig shows the distribution of RF titers in the general population.

## Discussion

We analyzed NHANES data for autoantibodies that are directly related to commonly occurring autoimmune conditions, such as rheumatoid arthritis, autoimmune thyroid disease, autoimmune diabetes, and celiac disease. The resulting estimates showed a substantial population-level prevalence of detectable serum autoantibodies among adults of all ages and for both women and men. In NHANES III (Table 5), 10% of younger adults ages 20–29 years and 18% of all adults had a detectable thyroid autoantibody—the latter representing an estimated 31 million persons in the US at that time. An estimated 32% of adults 60 years of age and older, or 12.8 million individuals, had at least one of the four measured autoantibodies RF, anti-TG, anti-TPO, or anti-TTG. Thus, the NHANES data, although based on a limited number of autoantibodies, directly challenge assertions that autoantibodies are uncommon. In retrospect, these findings should not come as a surprise because, historically, both at the inception of early humoral autoimmunity studies and periodically thereafter, small-scale surveys using differing laboratory methodologies have shown similar results [71–73].

The NHANES III thyroid autoantibody data in Tables 2 and 5 directly challenge the thesis that autoantibodies are seen most commonly in younger adults. To be sure, the NHANES data demonstrate that autoantibodies are common in younger adults, but the data also show that, overall, autoantibody prevalence increases significantly with age. In fact, the combined RF, anti-TG, anti-TPO, and anti-TTG data (Table 5) disclose that the oldest age groups have remarkably high autoantibody prevalence: in adults 60+ years of age in NHANES III, almost 1 in 3 had at least one detectable autoantibody.

Our findings also challenge the assertion that serum autoantibodies are primarily seen in women. Although we did find higher levels of autoantibodies among women, this is a relative difference since the prevalence of autoantibodies in both sexes is substantial. For example, among adults 60+ years of age, an estimated 39% of women and 22% of men had detectable autoantibodies. However specific autoantibodies tested here (RF, anti-TTG, anti-EMA) appeared to have no sex differential. Also, no sex differential is seen in Type 1 autoimmune diabetes in western countries [74]. Our intent here is not to detract from the importance of autoantibodies in women, but rather to emphasize that it is important to maintain a similar level of clinical suspicion in women and men with respect to the possibility of humoral autoimmunity.

Two caveats should be mentioned with respect to interpreting our results. First, although demographic variables, such as age and sex, are useful to characterize the population-level public health burden of autoantibodies, they are not explanatory variables for autoantibody occurrence. For example, we cannot conclude that age trends in the NHANES data are evidence that autoantibodies are a result of normal aging. The majority of younger and older adults have no detectable autoantibodies. Specific autoantibodies studied here show no age trends, and it remains to be determined whether other clinically important autoantibodies not studied here show any population-based age-related trends. A cautionary note here is that cumulative exposures to a specific causal risk factor can easily produce what appear to be age trends in data; a familiar example is tobacco smoking and the risk of chronic obstructive pulmonary disease or lung cancer. Second, although we emphasize here the high overall prevalence of autoantibodies, it is important not to dismiss as inconsequential the autoantibody population prevalence estimates in the range of one-half of one percent (0.5%) (e.g., anti-TTG, anti-EMA). In population-level studies, these seemingly low prevalences represent large numbers of individuals; for example, a 0.5% US national prevalence is equivalent to approximately 800,000 adults of all ages and 200,000 adults aged 60+ years. If such low-prevalence autoantibodies have important clinical consequences, the burden for patients and on the medical care system becomes substantial.

A precise estimate of the current US population prevalence of humoral autoimmunity is not possible here because NHANES did not test a full range of serum autoantibodies (see Limitations section below). Hence, our current estimates, by definition, are an undercount of true US population autoantibody prevalences. RF was not measured in NHANES 1999–2016, so a more current overall US prevalence estimate cannot be provided. However, if current RF prevalences are similar to NHANES III estimates, then the current overall population autoantibody prevalences for the four autoantibodies would be similar to but incrementally lower than the NHANES III estimates because of lower current thyroid autoantibody prevalences in the NHANES 2007–2012 data. However, if additional autoantibodies were added, current overall US autoantibody prevalences would equal or exceed the NHANES III estimates presented here. For example, anti-ENA antibody would contribute another 1% to overall US humoral autoimmunity estimates. This percentage might be higher because, in NHANES laboratory testing protocols, anti-ENA was tested only when ANA immunofluorescence signal strength was 3+ or 4+, and important autoantibodies, such as anti-Ro and anti-Su, do not show strong immunofluorescence. Further, anti-EMA testing was performed only when anti-TTG was positive. In addition, if anti-GAD65 data were available for current NHANES, it would likely add another 1–2% to overall US autoantibody prevalence. Finally, a variety of other autoantibodies currently under clinical investigation would add significantly to this listing [75].

It is beyond the scope of this report to address the clinical and public health implications of having autoantibodies. We note, however, that the prevalence estimates here are based primarily on target organ autoantibodies with well-known autoimmune disease associations. Other autoantibody-disease associations are typically vetted through extensive clinical/epidemiological studies. That process has perhaps engendered a climate of skepticism or even negativism regarding newly proposed autoantibody-disease associations. Certainly, all new autoantibody-disease associations should be thoroughly vetted before being accepted; however, the current lack of high-quality data for new autoantibody hazards should not be grounds for not continuing to investigate new clinical associations of autoantibodies as improved methods and databases become available. On a longer time-scale, i.e. decades, new autoantibody hazards will continue to be recognized on a regular basis, e.g., the new antibodies that recognize citrullinated proteins, such as anti-cyclic citrullinated peptide. Novel disease-specific autoantibodies will be discovered, and new health hazards of known autoantibodies will be identified. For example, the extra-articular manifestations of RF-associated diseases such as rheumatoid arthritis are increasingly well documented, especially in the lung, as are the extra-thyroidal manifestations of autoimmune thyroid disease [76, 77]. Other new associations include the association of rheumatoid arthritis autoantibodies with intrinsic lung disease, the long-term pulmonary mortality risks associated with IgA anti-TTG, and the possible trans-placental effects of thyroid autoantibodies [76–81]. These discoveries of new health outcome associations should be expected, as autoimmune diseases have diverse, protean manifestations, such as the wide-ranging clinical manifestations of systemic lupus erythematosus, celiac disease, dermatomyositis, Sjogren’s syndrome, and multiple sclerosis, among others [12, 13].

Scientifically, it is important to be clear about the logic of autoantibody health risks. Autoantibodies can be pathogenic, benign, or even helpful in some instances. However, once a definite example proving a specific autoantibody-related pathology is demonstrated or an autoantibody is shown to be significantly associated with increased risk of disease, it is a major scientific undertaking to rule out the possibility of additional types of adverse autoantibody-related health outcomes for that autoantibody. This requires a series of large, adequately powered studies covering the wide variety of possibilities for adverse clinical outcomes [82]. Absent this, when an autoantibody has already been shown to be associated with an adverse health outcome, there can be no warranty of safety against additional health effects until adequate research studies rule out those possibilities. Currently, the literature amply demonstrates autoantibody-related pathogenicity for many autoantibodies (i.e., proof of hazard) [2–4], and both low-level positive and high-level positive autoantibodies can be associated with adverse clinical outcomes [68, 83, 84]. Although this body of research is extensive, due to historical funding limitations, it is far from complete in any particular area. To our knowledge, there has never been a planned and executed program to screen even a single serum autoantibody against a wide range of potential health hazards. Thus, a significant level of ongoing clinical and Public Health concern regarding autoantibody safety is warranted.

## Study limitations

This study did not attempt to adjust the observed autoantibody prevalence estimates for the effects of immunodeficiency, immunosuppressive medications, or nutritional deficiencies. Adjustments for those factors would likely significantly increase the overall autoantibody prevalence estimates reported here. NHANES has such data available, but their analysis is beyond the scope of the current report. The prevalence estimates presented here are from cross-sectional data. It is known from clinical studies that in persons with specific autoimmune diseases, autoantibody levels can vary over time with the phase and treatment of the disease. Autoantibodies can be detected in the prodrome of clinical disease, and the highest autoantibody levels often primarily in early or highly active disease phases as opposed to later, treated disease or disease in clinical remission [85–88]. Also, as shown by the example of celiac disease, autoantibodies can rapidly revert to normal after cessation of a causal exposure. It follows that population-level cumulative lifetime autoantibody prevalences are likely to be substantially greater than the cross-sectional estimates portrayed here.

The NHANES autoantibody data that we used were collected as a series of independent studies rather than as an overall planned effort. Many important autoantibodies of known clinical consequence have not been studied by NHANES and NHANES does have the capability for studying these. Adding such data could substantially increase overall US autoantibody prevalence estimates. Table 6 presents selected examples of major disease-related autoantibodies thus far not studied by NHANES. The NHANES survey has a primary public health objective to address diabetes, cardiovascular disease, anemia, thyroid disease, and the health of older Americans. Remarkably, thus far only a single, partial-sample study of one diabetes autoantibody in adults was performed in NHANES; and no diabetes autoantibody data have been collected for the adolescent and pediatric population. Further, some proportion of individuals who are positive for red cell autoantibodies have related diagnosable disorders [89]. Thus far in NHANES, the two major pernicious anemia-autoimmune atrophic gastritis–related autoantibodies (anti-intrinsic factor autoantibody and anti-parietal cell autoantibodies) have not been studied. Undiagnosed, early, or preclinical pernicious anemia is estimated to be present in about 2–4% of Americans aged 60 and over, and early diagnosis and timely treatment can prevent dementia and other neurodegenerative outcomes [90, 91]. A final limitation is that cell-mediated autoimmunity might be a more significant cause of autoimmune disease than humoral autoimmunity; however, the NHANES survey has not collected data in this area. Lastly, NHANES has a limited amount of autoantibody data for adolescents aged 12–17 years and did not collect pediatric autoantibody data, even though these groups are affected by many important autoimmune conditions.

**Table 6:** Major clinically significant autoantibodies not studied in NHANES.

Autoantibodies	Disease Associations
Anti-Citrullinated Protein antibodies	Rheumatoid Arthritis
High-affinity Rheumatoid Factor	Rheumatoid Arthritis
Anti-single and double stranded DNA antibodies	Systemic Lupus Erythematosus
Anti-Histone antibodies	Drug-Induced Systemic Lupus Erythematosus
Anti-Cardiolipin; Lupus Anticoagulant; β2-Glycoprotein 1 antibodies	Anti-Phospholipid Syndrome, Stroke, Pulmonary Embolism
Anti-Myeloperoxidase, anti-Proteinase-3, anti-Neutrophil Cytoplasmic antibodies	Systemic Vasculitis syndromes
Islet-Cell Cytoplasmic, Insulinoma 2-Associated and anti-Insulin antibodies	Autoimmune Diabetes
Anti-Intrinsic Factor autoantibody, anti-Parietal Cell antibodies	Pernicious Anemia, Dementia, Neurodegenerative Disease
Anti-Mitochondrial antibodies	Primary Biliary Cholangitis
Anti-Red Cell antibodies	Autoimmune Hemolytic Anemia
Anti-Platelet antibodies	Immune Thrombocytopenia, bleeding disorders

An aim for future research is to support population-level screening for autoimmune disease risks in order to diagnose treatable disease earlier. However, that goal requires further research and cannot be achieved without an adequate, unbiased evidence base [92]. The NHANES data are a gold standard for population health research: they represent large nationally representative datasets from well-designed, professionally fielded health surveys that were designed to control for and minimize the effects of selection bias [51]. The methodological strengths of the NHANES surveys are their nationally representative samples, their high survey response rates, the over-sampling of older persons, and standardized, quality control–driven data collection protocols. The existing NHANES autoantibody datasets combined with the wealth of other NHANES laboratory and health examination data are a publicly available platform that can address many scientific questions relating to humoral autoimmunity. Also, additional new autoimmunity studies can be fielded as a part of the ongoing NHANES data collections. Finally, NHANES maintains stored sera suitable for autoantibody studies and has a capability for genetic and mortality follow-up research studies [93–96].

## Conclusions

Our findings provide US national estimates for the overall prevalence of autoantibodies in adults. Both target organ-specific and systemic disease autoantibodies are common in the US population. Although the NHANES data show trends for greater autoantibody prevalence with older age and among women, on a national level there are substantial autoantibody prevalences at all ages and among both women and men. Although adults of all ages have significant autoantibody burden, generally, older adults have the highest serum autoantibody prevalences. These results suggest that greater emphasis needs to be placed on understanding the true national-level scope and impact of humoral autoimmunity in order to provide a more reliable basis for clinical practice, disease prevention, and public health messaging.

## Supporting information

S1 Table Population prevalences for single (isolated) and multiple autoantibodies. This table provides estimates for the US population prevalences of a specific autoantibody as an isolated finding unaccompanied by any others, as well as the prevalences of persons having multiple autoantibodies, i.e. the various possible autoantibody combinations of Rheumatoid Factor, anti-TG, anti-TPO and anti-TTG taken two, three or four at a time. The list of possible combinations of the four autoantibodies is presented in the first column and the next set of columns to the right present the actually observed prevalence data and US population estimates for each of the autoantibody combinations.
(DOCX)

S2 Table Age-Specific autoantibody prevalence by sex. The analysis in the main paper presents age-specific and sex specific prevalences separately. Theoretically autoantibody age prevalences potentially could be non-uniform by sex, i.e. one sex might have a strong age-related prevalence trend and the other not. A thorough published analysis of the NHANES III data shows that this is not the case for the thyroid autoantibodies anti-TG and anti-TPO, the most prevalent autoantibodies in this study [45]. **S2 Table** below shows this is also not the case for RF.
(DOCX)

S3 Table Prevalence estimates for higher autoantibody levels. The literature often emphasizes high levels of autoantibodies seen in clinically active autoimmune disease, however both low and high level autoantibodies may have diagnostic and/or prognostic value. For example, as reviewed in the main paper Discussion section, “low positive” RF is associated with long-term mortality in RA patients and in current criteria, “low positive” RF levels have value for classifying symptomatic persons as having Rheumatoid Arthritis [68]. Also, in clinical practice, the current standard is to use the presence or absence of a detectable thyroid autoantibody, along with clinical signs and symptoms, to make diagnostic and therapeutic decisions [97]. The following tables present prevalence estimates for higher level RF and thyroid autoantibodies. Although the main analysis of the current paper is based on detectable serum autoantibodies, a sizeable fraction of the NHANES autoantibody data is in fact in higher ranges. The tables presented below show that for RF in the NHANES III data adults 60 + years, almost 70% of positive RF samples were greater than three times the detection limit. Similarly, using arbitrary 95^th^ percentile cut point criteria, in NHANES III data for US adults 18+ years, 25% had high anti-TG levels and 30% had high anti-TPO levels.
(DOCX)

S1 Fig The Distribution of positive rheumatoid factor titers in the general population. (TIF)

## Abbreviations

95% CI: 95% confidence interval
AID: autoimmune disease
ANA: antinuclear autoantibodies
anti-EMA: anti-endomysial autoantibodies
anti-ENA: antibodies to extractable nuclear antigens
anti-GAD65: autoantibodies to the 65-kDa isoform of glutamic acid decarboxylase
anti-TG: anti-thyroglobulin autoantibodies
anti-TPO: anti-thyroperoxidase autoantibodies
anti-TTG: anti-tissue transglutaminase
ELISA: enzyme-linked immunosorbent assay
NHANES: National Health and Nutrition Examination Surveys
NHES: National Health Examination Survey
RF: rheumato*i*d factor
RSE: relative standard error
US: United States
