# Supplementary material for: Population-based estimates of humoral autoimmunity from the U.S. National Health and Nutrition Examination Surveys, 1960–2014
Source: PLoS One. 2020 Jan 13;15(1):e0226516. doi: 10.1371/journal.pone.0226516 (PMC6957172; doi:10.1371/journal.pone.0226516)
Supplement: S1 Table — This table provides estimates for the US population prevalences of a specific autoantibody as an isolated finding unaccompanied by any others, as well as the prevalences of persons having multiple autoantibodies, i.e. the various possible autoantibody combinations of Rheumatoid Factor, anti-TG, anti-TPO and anti-TTG taken two, three or four at a time. The list of possible combinations of the four autoantibodies is presented in the first column and the next set of columns to the right present the actually observed prevalence data and US population estimates for each of the autoantibody combinations. (DOCX) [file pone.0226516.s001.docx]

**S1 Table. Autoantibody Prevalence Distributions: U.S. Population Ages 60+ Years,
NHANES III 1988-94**

| **Total Sample N=4,243** | **n** | **%** | **95% CI** | **US Census Population** |
| --- | --- | --- | --- | --- |
| ***Any Positive AutoAB******* | 1,659 | 31.9 | 30.3-33.5 | 12,792,282 |
| RF | 136 | 4.7 | 4.0-5.6 | 1,884,756 |
| anti-TG | 535 | 4.5 | 3.8-5.4 | 1,804,554 |
| anti-TPO | 658 | 7.1 | 6.3-7.9 | 2,847,185 |
| anti-TTG | 30 | 1.3 | 0.8-2.1 | 521,316 |
| anti-TG & TPO | 161 | 12.5 | 11.3-13.8 | 5,012,650 |
| ***2 Ab Combinations*** |  |  |  |  |
| anti-TG & TTG | 13 | 0 | * | * |
| anti-TG & RF | 47 | 0.3 | * | * |
| anti-TPO & TTG | 13 | 0.1 | * | * |
| anti-TPO & RF | 51 | 0.6 | 0.4-0.8 | 240,607 |
| anti-TTG & RF | 4 | 0.1 | * | * |
| ***3 Ab Combinations*** |  |  |  |  |
| anti-TG, TPO, & TTG | 4 | 0 | * | * |
| anti-TG, TPO, & RF | 9 | 0.8 | 0.5-1.3 | 320,810 |
| anti-TG, TTG, & RF | < 1 | 0 | * | * |
| anti-TPO, TTG, & RF | < 1 | 0 | * | * |
| ***4 Ab Combination*** |  |  |  |  |
| anti-TG, TPO, TTG, & RF | < 1 | 0 | * | * |
| ***All Ab Negative*** | 2,584 | 68.1 | 66.5-69.7 | 27,308,915 |

** For reference. *Variance estimate not statistically reliable for estimation, confidence intervals, and US Census

Population Totals not shown. Abbreviations: n= number of cases; 95%CI= 95% confidence interval.

Notes: Overall N=4,243- sample with complete data for antibodies tested. Antibody prevalence estimates are age

adjusted to the US population 60+ years 1989-1994 (total population estimate 40,101,198).
