# Supplementary material for: Population-based estimates of humoral autoimmunity from the U.S. National Health and Nutrition Examination Surveys, 1960–2014
Source: PLoS One. 2020 Jan 13;15(1):e0226516. doi: 10.1371/journal.pone.0226516 (PMC6957172; doi:10.1371/journal.pone.0226516)
Supplement: S2 Table — The analysis in the main paper presents age-specific and sex specific prevalences separately. Theoretically autoantibody age prevalences potentially could be non-uniform by sex, i.e. one sex might have a strong age-related prevalence trend and the other not. A thorough published analysis of the NHANES III data shows that this is not the case for the thyroid autoantibodies anti-TG and anti-TPO, the most prevalent autoantibodies in this study [45]. S2 Table below shows this is also not the case for RF. (DOCX) [file pone.0226516.s002.docx]

| **S2 Table. Rheumatoid Factor by Sex and Age, NHANES Surveys** | | | | |
| --- | --- | --- | --- | --- |
| **a. NHES I 1960-1962** | **N** | **RF Positive** | **%** | **95% CI** |
| **Males** |  |  |  |  |
| 18-24 Years | 405 | 3 | 0.5 | * |
| 25-39 Years | 987 | 19 | 1.9 | 1.0-2.9 |
| 40-59 Years | 1095 | 34 | 4.0 | 2.4-5.6 |
| 60+ Years | 516 | 20 | 3.9 | 2.0-5.9 |
| **Females** |  |  |  |  |
| 18-24 Years | 515 | 6 | 0.9 | * |
| 25-39 Years | 1136 | 20 | 1.7 | 0.9-2.6 |
| 40-59 Years | 1269 | 42 | 3.2 | 1.8-4.7 |
| 60+ Years | 545 | 41 | 7.4 | 5.3-9.6 |
| **b. NHANES III 1989-1994** | **N** | **RF Positive** | **%** | **95% CI** |
| **Males** |  |  |  |  |
| 60-69 Years | 1,126 | 53 | 4.0 | 2.7-6.0 |
| 70+ Years | 1431 | 116 | 8.2 | 6.4-10.4 |
| **Females** |  |  |  |  |
| 60-69 Years | 1,099 | 84 | 6.4 | 5.0-8.2 |
| 70+ Years | 1,614 | 125 | 7.4 | 5.9-9.2 |

*Variance estimate not statistically reliable. N= Total sample size.
