# Supplementary material for: Population-based estimates of humoral autoimmunity from the U.S. National Health and Nutrition Examination Surveys, 1960–2014
Source: PLoS One. 2020 Jan 13;15(1):e0226516. doi: 10.1371/journal.pone.0226516 (PMC6957172; doi:10.1371/journal.pone.0226516)
Supplement: S3 Table — The literature often emphasizes high levels of autoantibodies seen in clinically active autoimmune disease, however both low and high level autoantibodies may have diagnostic and/or prognostic value. For example, as reviewed in the main paper Discussion section, “low positive” RF is associated with long-term mortality in RA patients and in current criteria, “low positive” RF levels have value for classifying symptomatic persons as having Rheumatoid Arthritis [68]. Also, in clinical practice, the current standard is to use the presence or absence of a detectable thyroid autoantibody, along with clinical signs and symptoms, to make diagnostic and therapeutic decisions [97]. The following tables present prevalence estimates for higher level RF and thyroid autoantibodies. Although the main analysis of the current paper is based on detectable serum autoantibodies, a sizeable fraction of the NHANES autoantibody data is in fact in higher ranges. The tables presented below show that for RF in the NHANES III data adults 60 + years, almost 70% of positive RF samples were greater than three times the detection limit. Similarly, using arbitrary 95th percentile cut point criteria, in NHANES III data for US adults 18+ years, 25% had high anti-TG levels and 30% had high anti-TPO levels. (DOCX) [file pone.0226516.s003.docx]

| **S3 Table. Prevalence Estimates for Higher Autoantibody Levels.** | | | | |
| --- | --- | --- | --- | --- |
| **a. Anti-Thyroglobulin Autoantibody ≥ 58 IU/ml (95^th^ percentile). NHANES III 1988-1994.** | | | | |
|  | **N** | **n** | **%** | **95%CI** |
| **Overall Prevalence** | 15,956 | 471 | 3.2 | 2.7-3.7 |
| **Age Groups (years)** |  |  |  |  |
| 18-24 | 2,286 | 36 | 1.2 | ***** |
| 25-39 | 4,674 | 79 | 2.4 | 1.5-3.2 |
| 40-59 | 4,161 | 114 | 3.5 | 2.7-4.3 |
| 60-74 | 3,126 | 131 | 5.0 | 3.8-6.2 |
| 75+ | 1,709 | 111 | 6.8 | 5.3-8.3 |
| **Males** | 7,471 | 128 | 1.9 | 1.4-2.4 |
| **Females** | 8,485 | 343 | 4.4 | 3.7-5.1 |

*Variance estimate not statistically reliable. N=total sample; n=number of cases.

| **b. Anti-Thyroperoxidase Autoantibody ≥ 13.5 IU/ml (95th percentile).** | | | | |
| --- | --- | --- | --- | --- |
|  | **N** | **n** | **%** | **95%CI** |
| **Overall Prevalence** | 15,956 | 641 | 4.3 | 3.9-4.8 |
| **Age Groups (years)** |  |  |  |  |
| 18-24 | 2,286 | 38 | 1.3 | ***** |
| 25-39 | 4,674 | 165 | 4.0 | 3.0-4.9 |
| 40-59 | 4,161 | 192 | 5.2 | 4.2-6.2 |
| 60-74 | 3,126 | 159 | 5.9 | 4.7-7.1 |
| 75+ | 1,709 | 87 | 5.1 | 3.8-6.5 |
| **Males** | 7,471 | 143 | 2.2 | 1.7-2.7 |
| **Females** | 8,485 | 498 | 6.3 | 5.4-7.2 |

*Variance estimate not statistically reliable. N=total sample; n=number of cases.

| **c. Rheumatoid Factor ≥ 1:128 (>3X detection limit). NHES I 1960-1962.** | | | | |
| --- | --- | --- | --- | --- |
|  | **N** | **n** | **%** | **95%CI** |
| **Overall Prevalence** | 6,468 | 62 | 1.1 | 0.7-1.4 |
| **Age Groups (years)** |  |  |  |  |
| 18-34 | 2,306 | 6 | 0.2 | ***** |
| 35-44 | 1,448 | 14 | 1.2 | * |
| 45-59 | 1,653 | 20 | 1.5 | 0.7-2.3 |
| 60+ | 1,061 | 22 | 2.0 | 0.9-3.0 |
| **Males** | 3,003 | 28 | 1.0 | 0.6-1.4 |
| **Females** | 3,465 | 34 | 1.1 | 0.5-1.7 |

*Variance estimate not statistically reliable. N=total sample; n=number of cases.

| **d. Rheumatoid Factor ≥ 1:160 (>3X detection limit). NHANES III 1988-1994.** | | | | |
| --- | --- | --- | --- | --- |
|  | **N** | **n** | **%** | **95%CI** |
| **Overall Prevalence** | 5,270 | 255 | 4.4 | 3.7-5.1 |
| **Age Groups (years)** |  |  |  |  |
| 60-69 | 2,225 | 91 | 1.9 | 1.4-2.4 |
| 70+ | 3,045 | 164 | 2.5 | 2.0-3.0 |
| **Males** | 2,558 | 110 | 1.6 | 1.2-2.0 |
| **Females** | 2,713 | 145 | 2.9 | 2.3-3.5 |

N=total sample; n=number of cases.
