# Supplementary figures and images for: Population-based estimates of humoral autoimmunity from the U.S. National Health and Nutrition Examination Surveys, 1960–2014
Source: PLoS One. 2020 Jan 13;15(1):e0226516. doi: 10.1371/journal.pone.0226516 (PMC6957172; doi:10.1371/journal.pone.0226516)

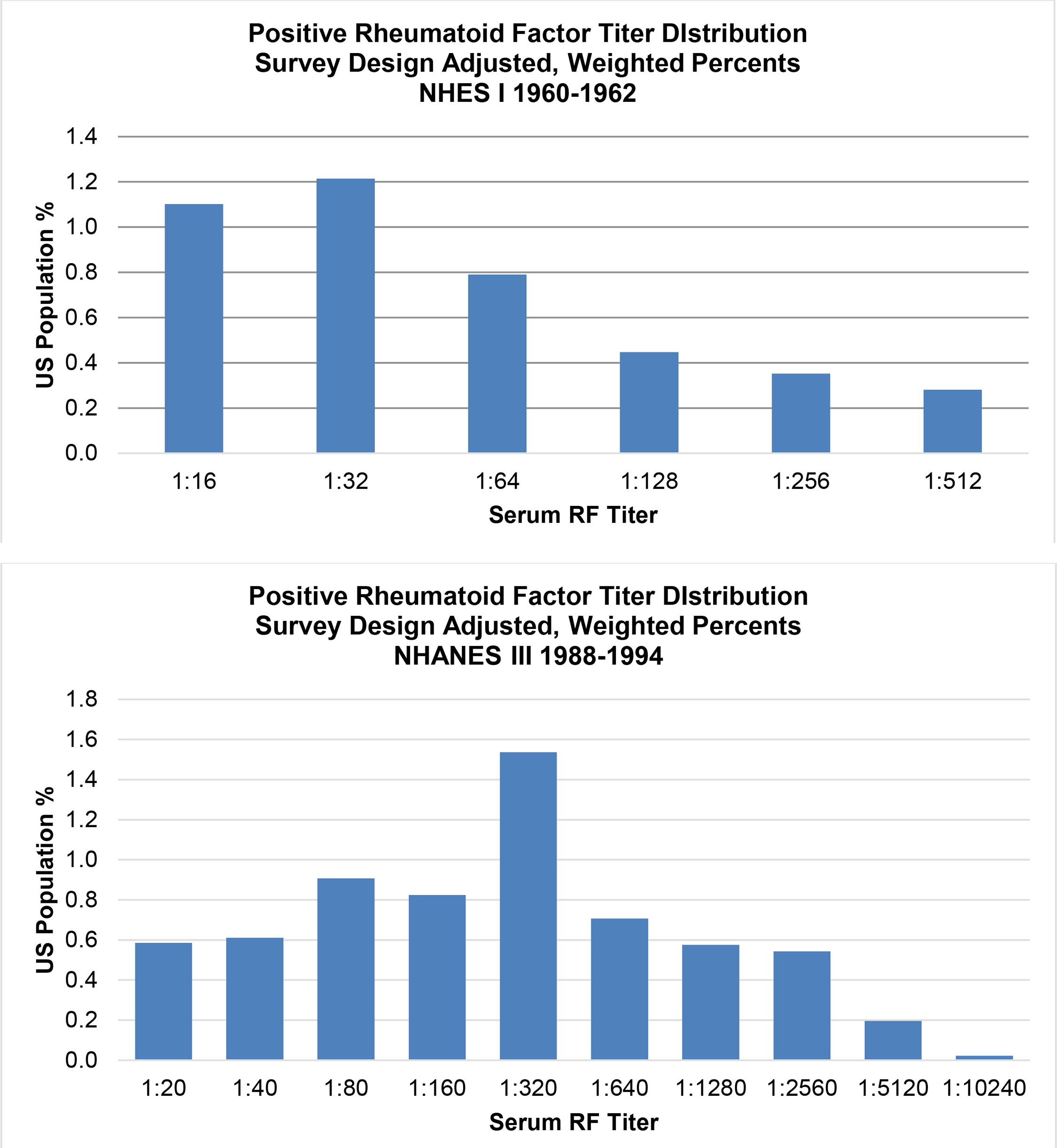

Supplement: S1 Fig — (TIF) [file pone.0226516.s004.tif]
